# Two and Three-Dimensional Echocardiography in Primary Mitral Regurgitation: Practical Hints to Optimize the Surgical Planning

**DOI:** 10.3389/fcvm.2021.706165

**Published:** 2021-07-08

**Authors:** Maria Concetta Pastore, Giulia Elena Mandoli, Anna Sannino, Aleksander Dokollari, Gianluigi Bisleri, Flavio D'Ascenzi, Luna Cavigli, Annalisa Pasquini, Matteo Lisi, Nicolò Ghionzoli, Ciro Santoro, Marcelo Haertel Miglioranza, Marta Focardi, Giuseppe Patti, Serafina Valente, Sergio Mondillo, Matteo Cameli

**Affiliations:** ^1^Division of Cardiology, Department of Medical Biotechnologies, University of Siena, Siena, Italy; ^2^Maggiore della Carità Hospital, University of Eastern Piedmont, Novara, Italy; ^3^Department of Advanced Biomedical Science, Federico II University Hospital Naples, Naples, Italy; ^4^Baylor Research Institute, The Heart Hospital Baylor Plano, Plano, TX, United States; ^5^Cardiac Surgery, St. Michael Hospital, Toronto, ON, Canada; ^6^Department of Cardiovascular and Thoracic Sciences, Fondazione Policlinico Universitario A. Gemelli, Istituto di Ricovero e Cura a Carattere Scientifico (IRCCS), Università Cattolica del Sacro Cuore, Rome, Italy; ^7^Division of Cardiology, Department of Cardiovascular Diseases – Azienda Unità Sanitaria Locale (AUSL) Romagna, “Santa Maria delle Croci” Hospital, Ravenna, Italy; ^8^Cardiology Institute of Rio Grande Do Sul, University Foundation of Cardiology, Porto Alegre, Brazil

**Keywords:** mitral regurgitation, echocardiography, three-dimensional, surgery, planning

## Abstract

Primary mitral regurgitation (MR) is the second most common valvular disease, characterized by a high burden in terms of quality of life, morbidity, and mortality. Surgical treatment is considered the best therapeutic strategy for patients with severe MR, especially if they are symptomatic. However, pre-operative echocardiographic evaluation is an essential step not only for surgical candidate selection but also to avoid post-operative complications. Therefore, a strong collaboration between cardiologists and cardiac surgeons is fundamental in this setting. A meticulous pre-operative echocardiographic exam, both with transthoracic or transesophageal echocardiography, followed by a precise report containing anatomical information and parameters should always be performed to optimize surgical planning. Moreover, intraoperative transesophageal evaluation is often required by cardiac surgeons as it may offer additive important information with different hemodynamic conditions. Three-dimensional echocardiography has recently gained higher consideration and availability for the evaluation of MR, providing more insights into mitral valve geometry and MR mechanism. This review paper aims to realize a practical overview on the main use of basic and advanced echocardiography in MR surgical planning and to provide a precise checklist with reference parameters to follow when performing pre-operative echocardiographic exam, in order to aid cardiologists to provide a complete echocardiographic evaluation for MR operation planning from clinical and surgical point-of-view.

## Introduction

Mitral regurgitation (MR) is the second most common valvular heart disease (after aortic stenosis), and the second most frequent indications for cardiac surgery. Primary MR consist of a mitral apparatus anatomic disease, with the most frequent etiology being degenerative.

The current European Society of Cardiology (ESC) guidelines recommend surgical treatment for MR in case of symptomatic patients with severe chronic primary MR, or asymptomatic with left ventricular (LV) dysfunction [LV enlargement or reduced ejection fraction (EF)], taking also into account the presence enlarged left atrium and/or of atrial fibrillation (AF) due to the risk of embolic events (47) or pulmonary hypertension (PH) (1).

American guidelines recommend mitral valve (MV) surgery for symptomatic patients with chronic severe primary MR or asymptomatic patients with LV dysfunction (assessed with LV EF 30–60% and/or LV end-systolic diameter >40 mm) (2).

However, when the patient has been referred for surgical treatment of MR, a meticulous planning of the intervention is the cornerstone for its success. At this stage, a close collaboration between the cardiologist and the cardiac surgeon is paramount to facilitate the surgical procedure and to ensure patient the best management.

Transthoracic and transesophageal echocardiography play a key role for the pre-operatory evaluation of MR patients. Beyond the conventional indices used for the assessment and quantification of MR (3), there are additional anatomic and functional measures of mitral valve apparatus which are highly required from cardiac surgeons before intervention. Therefore, each cardiac imager should be trained on the elements and parameters to focus on when evaluating a MR patient waiting for surgery, in order to operate a complete echocardiographic assessment and provide all the required information for this multidisciplinary approach. The present review aims to describe the pivotal elements for the correct planning of MR surgery analyzing the echocardiographer and cardiac surgeons' points of view, in order to assist clinicians in the use of traditional and newest echocardiographic techniques preoperative MR evaluation.

## 2D Echocardiography: Focus on Mitral Valve

The first task of the echocardiographer is to provide better insight on mitral valve (MV) anatomy, which can be divided into 6 scallops: 3 constituting the anterior leaflet, and 3 the posterior leaflet. Imagers' attention should be focused on different anatomic point depending on the various mechanisms of MR. The mostly known classification of primary MR etiology by Carpentier includes (4):

- Type 1-normal leaflet motion (leaflet perforation or cleft),
- Type II: excessive leaflet motion (leaflet prolapse),
- Type III-restricted leaflet motion (rheumatic MR, calcification, drug-induced MR)

Arguably, these primary anomalies of mitral valve anatomy and function and their different complexity will require different approaches for surgical treatment (5) (that would be further explained later). Accordingly, there will be some different paramount pre-operative information that could be provided by echocardiography [(6); Table 1].

**Table 1:** Preferred surgical treatment and necessary echocardiographic information according to Carpentiers' classification of primary mitral regurgitation, with representative cases for each type by transoesophageal echocardiography.

**Carpentiers' type**	**Examples**	**Surgical treatment**	**Echocardiographic information**
Type 1	Annulus dilation, Leaflet perforation or cleft	Annuloplasty	- Annulus dimensions (to confirm its dilation as mechanism of MR)
Normal leaflet motion 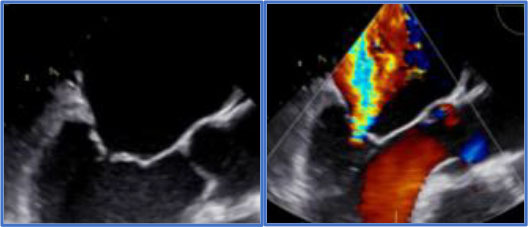		Pericardial patch repair	- Tricuspid annulus measure (prediction of residual functional TR)
Type 2	Chordal rupture	Gortex neo-chordae	- Accurately identify the scallops involved in the prolapse (multiplanar TEE)
MV prolapse 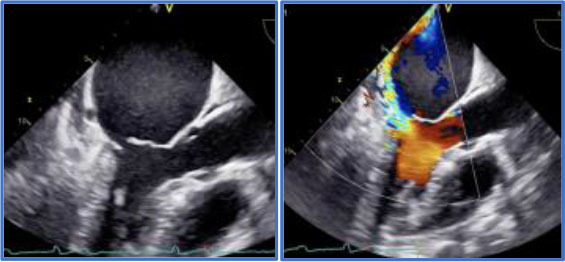	Chordal elongation	Chordal transfer	- Coaptation-septum distance and
	Papillary rupture	Triangular resection	Length of PL (to avoid post-operative SAM)
		Quadrangular resection Resection and sliding plasty (recommended if PL >2 cm)	- LV dimensions and EF
Type 3 Restricted leaflet motion 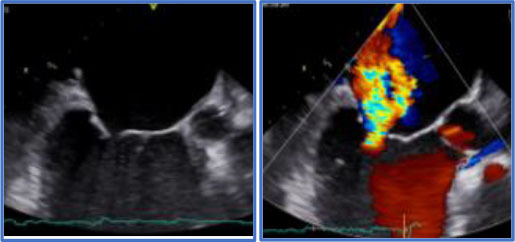	Restricted leaflet opening Commissural or chordal fusion Leaflet thickening Leaflet calcification	Chordal division	Identify affected chorda/ae Coaptation depth Tenting area
	Restricted leaflet closure Chordal thickening Chordal shortening	Annuloplasty	EROA

*EF, ejection fraction; EROA, effective regurgitant orifice area; LV, left ventricular; MR, mitral regurgitation; PL, posterior leaflet; TEE, transesophageal echocardiography; TR, tricuspid regurgitation*.

Beyond these essential parameters, the following MV anatomic measures are considered useful for guiding surgical techniques in each type of MR etiology (Supplementary Figure 1):

Annulus area

- Chordae and leaflet length
- Presence of calcification
- Anterior leaflet-IVS distance
- Mitral-aortic angle
- Interventricular septum (IVS) thickness and presence of subaortic spur
- Left ventricular outflow tract measure.

### Bidimensional Echocardiography: Overview on Cardiac Function

#### Left Heart

Transthoracic echocardiography (TTE) is the gold standard technique to assess LV dimension and function: not only LV dysfunction could be the cause of a secondary MR, but could be the consequence of primary MR, due to a chronic volume overload. For the quantification of LV volumes and function, both 2D (Simpson method) and 3D echocardiography are recommended (3).

It is known that LA enlargement is a common consequence of chronic MR. In fact, LA dimensions are considered in MR severity assessment since patients with smaller LA are less likely to have severe MR. LA volume should be measured by 2D echocardiography, with biplane Simpson disks method at end-systole (7).

**Figure 1:**
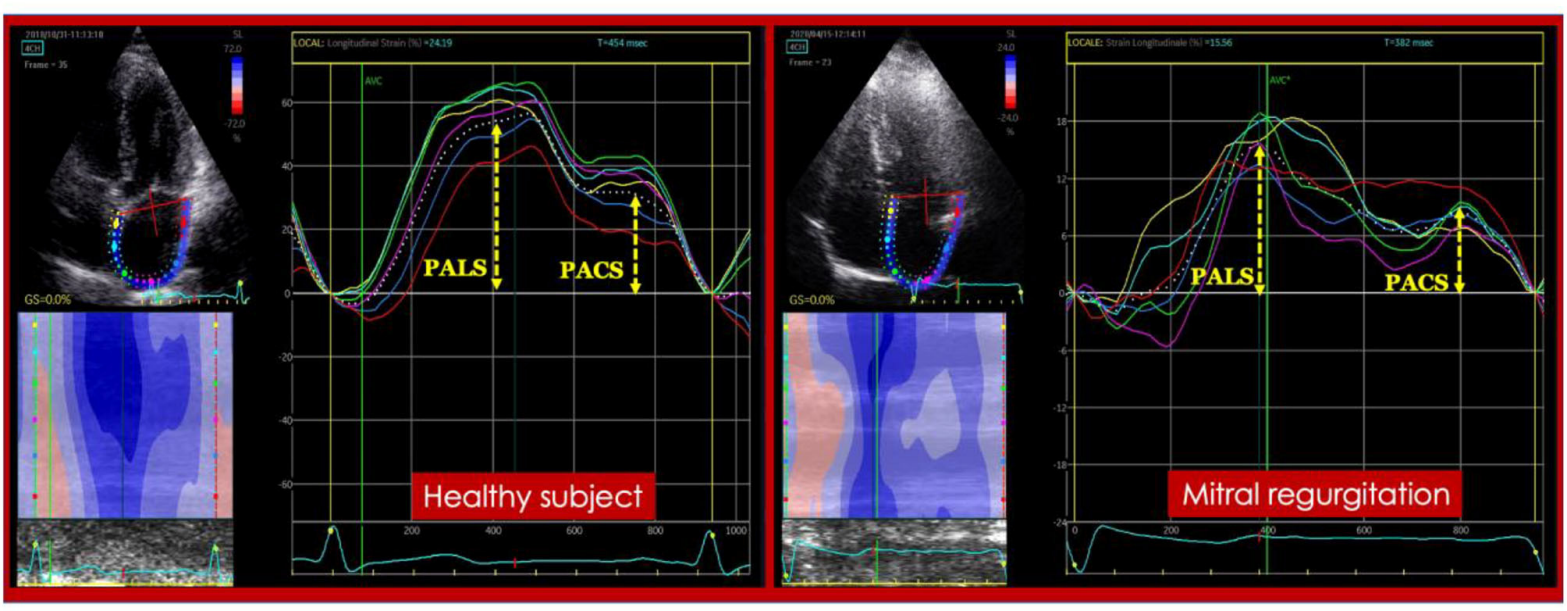
Left atrial strain in a healthy subject **(Left)** and in a patient with severe mitral regurgitation **(Right)**. PACS, peak atrial contraction strain; PALS, peak atrial longitudinal (reservoir) strain.

Recent evidence has shown how the use of advanced deformation imaging could provide early identification of left heart impairment in chronic MR and additive prognostic information. This could be easily and reliably assessed by speckle tracking echocardiography (8), with an offline analysis of 2D echocardiographic indices providing information on LV and LA longitudinal deformation all over the cardiac cycle, quantified as LV and LA longitudinal strain (this could be divided into LA reservoir; conduit and contractile strain based on LA deformation phases) (Figure 1). LA strain has been shown to describe LA worsening function parallel to MR severity in patients with asymptomatic MR (9), moreover, it correlates with invasively-assessed myocardial fibrosis caused by severe MR in patients with MV prolapse (10) and is a strong predictor of outcome not only in patients with severe MR (11, 12), but also in patients with moderate MR (13).

In a recent study by Kim et al., preoperative LV global longitudinal strain (GLS) showed a better prediction of cardiac outcome after surgery in 506 patients with severe MR compared to conventional parameters (LV dysfunction, AF, type of surgery) (14). Furthermore, LV GLS was an independent predictor of exercise capacity in 660 asymptomatic patients with >3 primary MR, preserved LV EF and non-dilated LV.

#### Right Heart

As shown in Table 1, the assessment of tricuspid anatomic properties and of coexisting tricuspid regurgitation (TR) is of outmost importance in pre-surgical evaluation of MR in order to assess the necessity of concomitant tricuspid annuloplasty during MV intervention. In fact, it has been demonstrated (15, 16) that the pre-operative tricuspid annulus measurement predicts residual functional TR post- MV surgery. Current recommendations for tricuspid valve surgery state that:

- Surgery is recommended in patients with severe TR who should undergo left-sided valve surgery regardless of symptoms (Class I),
- Surgery should be considered (Class IIa) in patients with mild/moderate secondary TR and/or significant annular dilatation (≥40 mm or 21 mm/m^2^; Class IIa) (1).

Moreover, a conscious assessment of RV dimensions and function would be important before MV surgery, since RV function could be considerably affected by cardiac surgery (15). On the other hand, pre-existing RV structural and functional damage could represent a higher risk of worse outcome after intervention (16) even if more evidence is required in this field. RV enlargement could be assessed by linear dimensions: basal diameter >42 mm and/or mid-cavity diameter >33 mm indicate RV dilatation; and RV areas (both end-diastolic and end-systolic area), that could also be used to calculate an index of RV function, RV fractional area change as (end-diastolic – end-systolic area)/end diastolic area. Also, M-mode tricuspid annular plane systolic excursion (<17 mm) and tricuspid annular systolic tissue Doppler velocity (<11 cm/s) are recommended as markers of RV dysfunction. STE could also be useful in these cases, in fact, RV strain has demonstrated its superiority over basic echocardiographic indices for the assessment of RV function, since, unlike the previous indices which analyze the annular portion of RV, it provides a more complete assessment of RV global longitudinal function and has also shown to correlate with RV pressures and outcome in patients with heart failure (17).

### When a Transesophageal Approach Is Necessary

Even though TTE could provide enough diagnostic and pre-operative information for patients with severe MR, multiplane transesophageal echocardiogram (TEE) is currently required in almost all cases of MR before surgery, especially in presence of suboptimal TTE image quality (7), in absence of absolute contraindications (Table 2). As TTE, TEE may assess all the required parameters, listed in in Table 3, using 4-chamber, inter-commissural, and long-axis views, with higher sensitivity than TTE alone, resulting in deeper insights into the precise mechanisms of MV disease and the precise definition of the site responsible for MV dysfunction [(20); Supplementary Figure 1].

**Table 2:** Absolute and relative contraindications to perform transoesophageal echocardiography [modified from (18)].

**Absolute contraindications**	**Relative contraindications**
Recent esophageal/gastric surgery	History of radiation to neck and mediastinum
Esophageal obstruction (stricture, tumor)	History of GI surgery
Esophageal perforation, laceration	Recent upper GI bleed
Esophageal diverticulum	Barrett's esophagus
Active upper GI bleed	History of dysphagia
Perforated viscus	Restriction of neck mobility (severe cervical arthritis, atlantoaxial joint disease)
	Symptomatic hiatal hernia
	Esophageal varices
	Coagulopathy, thrombocytopenia
	Active esophagitis
	Active peptic ulcer disease

*GI, gastro-intestinal*.

**Table 3:** Pre-operative echocardiographic checklist for planning mitral regurgitation surgery (6, 18, 19).

*Pre-operative checklist*		
Patient : ____________________________		
Type of Intervention : _____________________________		
Date : _____________________		
*Bidimensional Echocardiography:* transthoracic		
transesophageal		
**Parameter**	**Measure**	**Normal values**
**Anatomic measures**		
**Mitral annular dimension**		
- Diameter (PSAX *in diastole*)		<35 mm
- Annulus area		5–11 cm^2^
Annulus/anterior leaflet ratio		<1.3
Number of involved scallops		–
Calcification (++++ max.)		–
Coaptation point-to-septum distance		≥2.5 cm
Mitral-aortic angle (°)		136 ± 13 *end-diastole* 129 ± 11 *end-systole*
IVS thickness (mm)		<11 mm
Subaortic spur *(yes/no and mm)*		No
LVOT measure (mm)		20 ± 2
Leaflet length (mm)		
Anterior leaflet (AL)		≥26
Posterior leaflet (PL)		<15
AL/PL ratio		>1.3
PL angle (°)		<45
Chordae length (mm)		
Segments height (P1–P3, cm)		<1.5
Tenting area (cm^2^)		<2.5
Asymmetric tenting (yes/no)		
**Functional measures**		
LV EDD (mm)		42–59
LV EDD/BSA (mm/m^2^)		2.2–3
LV ESV (ml)		21–61
LV ESV/BSA (ml/m^2^)		11–31
LV EF (%)		55–60%
LV GLS (%)		≥-20%
LAVI (ml/m^2^)		16–34
PALS (%)		≥39%
fwRVLS (%)		≥-20%
**3D transesophageal echocardiography**		
VCA (cm^2^)		≤ 0.4 (cut-off for severe MR)
Presence of cleft/indentation		No
A2 height (mm)		≤ 26
P2 height (mm)		<20
Inter-trigonal distance (mm)		30 ± 3

*BSA, body-surface area; EDD, end-diastolic diameter; EF, ejection fraction; ESV, end-systolic volume; fwRVLS, free-wall right ventricular longitudinal strain; GLS, global longitudinal strain; IVS, interventricular septum; LAVI, left atrial volume index; LV, left ventricular; LVOT, left ventricular outflow tract; PALS, peak atrial longitudinal strain; PSAX, parasternal short axis; VCA, vena contracta area*.

Particularly, TEE would be useful to assess the feasibility of durable repair (21), and the signs of higher probability of treatment failure:

1. Anatomic characteristics (22):
  - Large central regurgitant jet
  - Severe annular dilatation (> 50 mm)
  - Involvement of ≥3 scallops
  - Extensive valve calcification

2. Risk factors for post-operative SAM (with hemodynamic instability) (7, 18, 19)
  - Coaptation-septum distance
  - Mitral-aortic angle
  - Long anterior leaflet (AL height at A2, middle scallop, to identify appropriate ring size)
  - Length of PLs and ratio between anterior and PL length
  - Non-dilated and hyper-dynamic LV

3. For Type 3 Carpentier severe regurgitant jet associated with (18):
  - MV annulus diameter ≥37 mm
  - Coaptation distance >1 cm
  - Systolic tenting area >2.5 cm^2^
  - PL angle> 45°
  - Presence of asymmetric tethering

Moreover, in the last years TEE has affirmed its pivotal role in intraoperative evaluation, in order to obtain a real-time assessment of the results of MV repair/replacement (e.g., residual regurgitant jet; presence of SAM), and improve surgical outcome.

However, TEE is not always promptly available, is an invasive method (sometimes requiring sedation), and has several absolute and relative contraindications (Table 2), therefore sometimes TTE should be preferred.

## Three-Dimensional Echocardiography

### Role in Pre-procedural Planning

The role of 3D echocardiography in the setting of MV surgery for primary MR can be summarized in 3 main objectives:

1. Assess MR severity by 3D vena contracta area (VCA) and/or 3D reconstruction modeling
2. Define the “reparability” of the MV
3. Sizing of the annuloplasty band or ring

In cases where 2D assessment of MR severity is doubtful, 3D echocardiography can help obtaining an accurate assessment of the severity of the regurgitation.

The introduction of 3D echocardiography into clinical practice has provided direct measurement of VCA. This could be obtained by multiplanar reconstruction tools to orient orthogonal imaging planes (x and y) through the long axis of the MR jet, with the z plane adjusted perpendicularly through the narrowest cross-sectional area of the vena contracta. VCA is then measured by manual planimetry of the color Doppler signal (Supplementary Figure 2). In case of MR characterized by multiple jets, the imaging plane should be oriented through each jet separately for tracing. A value of 3D VCA >0.4 cm^2^ denotes severe MR (23).

The reparability of a MV depends on two important considerations:

a. The presence of extensive leaflet or annular calcification;
b. The presence of a complex degenerative anatomy e.g., anterior MV leaflet prolapse, bileaflet prolapse, or Barlow disease (24).

**Figure 2:**
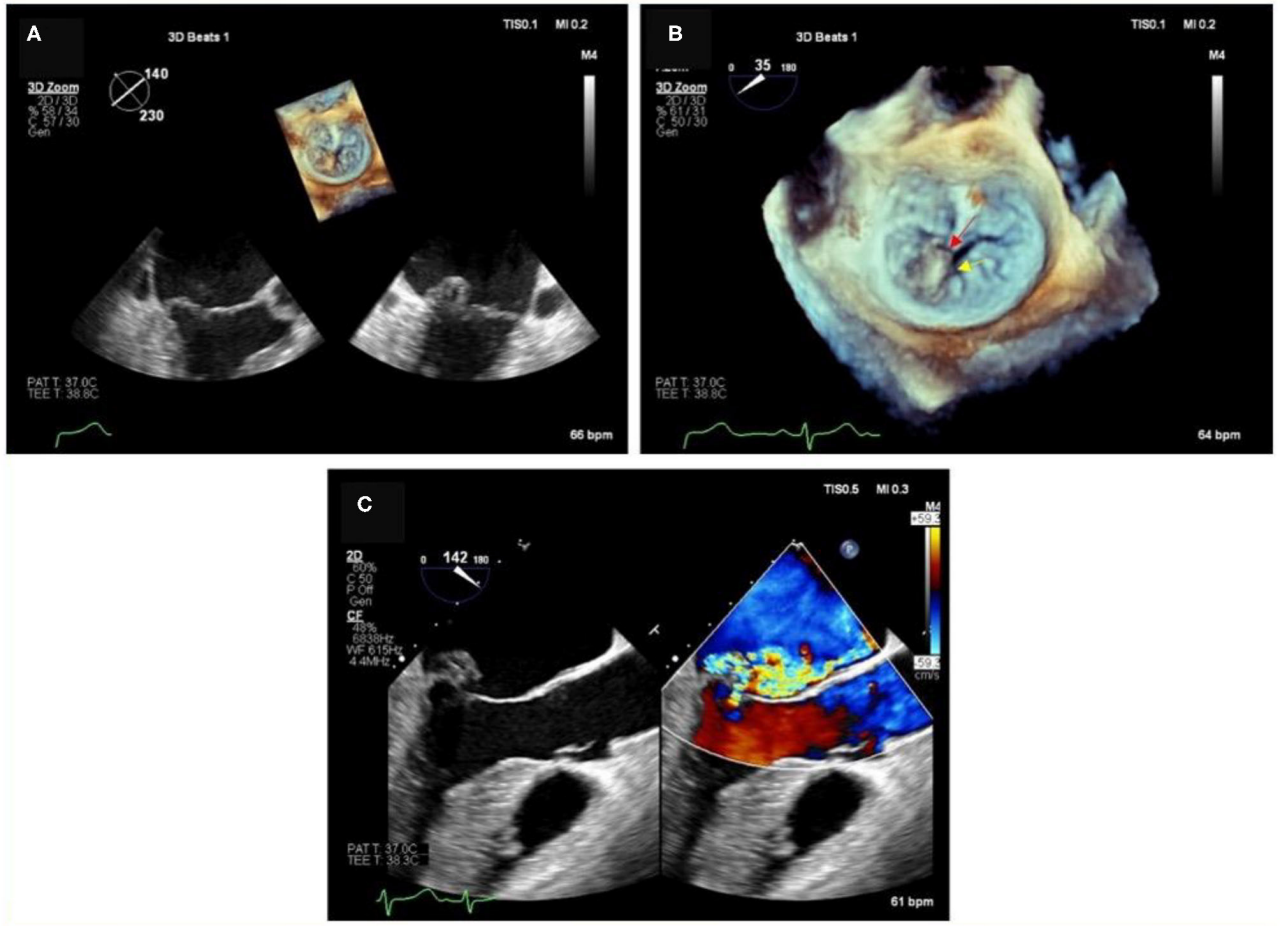
Degenerative MR due to P2 flail in the context of a complex anatomy. **(A)** 2D images and relative 3D (on top) showing P2 leaflet flail form the long-axis view and commissural view. **(B)** Magnification of the 3D short-axis on the MV (surgeons' view), where the P2 flail is easily appreciated (red arrow), together with a deep indentation (cleft- yellow arrow) between P2 and P3, which justify the presence of multiple jets. **(C)** Color Doppler image of the long-axis view, showing at least 2 regurgitant MR jets.

Significant annular calcification hampers the possibility and/or the durability of the repair. On the other hand, the presence of complex degenerative anatomy in the hands of low experienced surgeons may lead to suboptimal repair or MV replacement and also affects the durability of repair. The presence of clefts or indentations is as well very important as it contributes to the complexity of the disease and thus of the intervention. 3D Echocardiography and in particular 3D TEE are extremely helpful for the surgeon to identify such complex anatomies as well as to investigate the presence of indentations and clefts in the MV leaflets [(22, 24–27); Figure 2].

**Figure 3:**
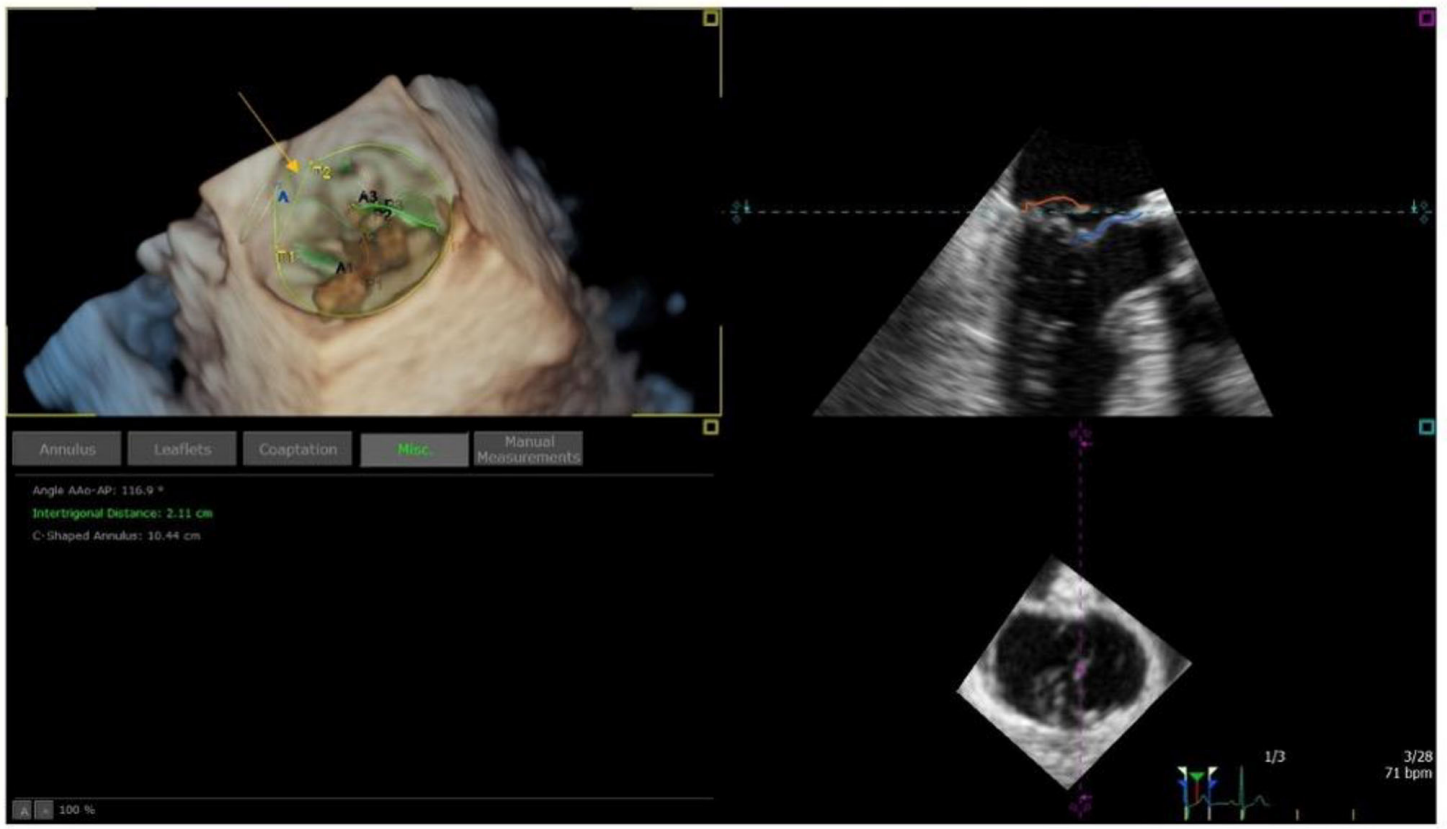
Example of MV model, used to estimate inter-trigonal distance (T1-T2 distance in the top left panel; orange arrow) and thus MV ring size.

Secondly, but not less important, quantitative 3D TEE allows to measure A2 height, inter-trigonal distance, and total annular perimeter size, which are useful to determine the size of the annuloplasty ring or band. In fact, A2 height and inter-trigonal distance are most commonly evaluated by surgeons during the operation. Despite the fact that 3D TEE does not replace the final sizing done by the surgeon in the OR, it does represent a useful guide as this information, if available before the procedure, could be useful for the surgeon to make comparisons to intraoperative sizing, which is done differently by different surgeons (28). The other quantitative data very helpful to the surgeon is the P2 height; if this is >20 mm, the reduction of this measure becomes essential to prevent post-operative SAM of the MV and LVOT obstruction [(29); Figure 3].

3D TEE may also have a pivotal role to accurately describing all MV lesions (e.g., clefts, commissural abnormalities, and tethering of the anterior MV leaflet) which are essential when planning chordal implantation, to ensure appropriate patient selection.

### Role in the Operating Room

Procedural guidance for surgical MV repair is developed around a comprehensive baseline intraoperative TEE to establish (or confirm) findings in the pre-preprocedural TEE and, above all, the anatomic basis of the MR. Moreover, assessment of LV and RV function, the evaluation of the degree of TR and measurement of the tricuspid annulus specifically are essential. Post-operative TEE evaluation includes locating and detecting intracardiac air when coming off cardiopulmonary bypass and assessment of the integrity of the MV repair. If residual MR is present, its severity and origin must be investigated. A post-procedural evaluation of the LV and RV function is also essential to ensure that there has not been a significant change from baseline.

## Conclusions

**Figure 4:**
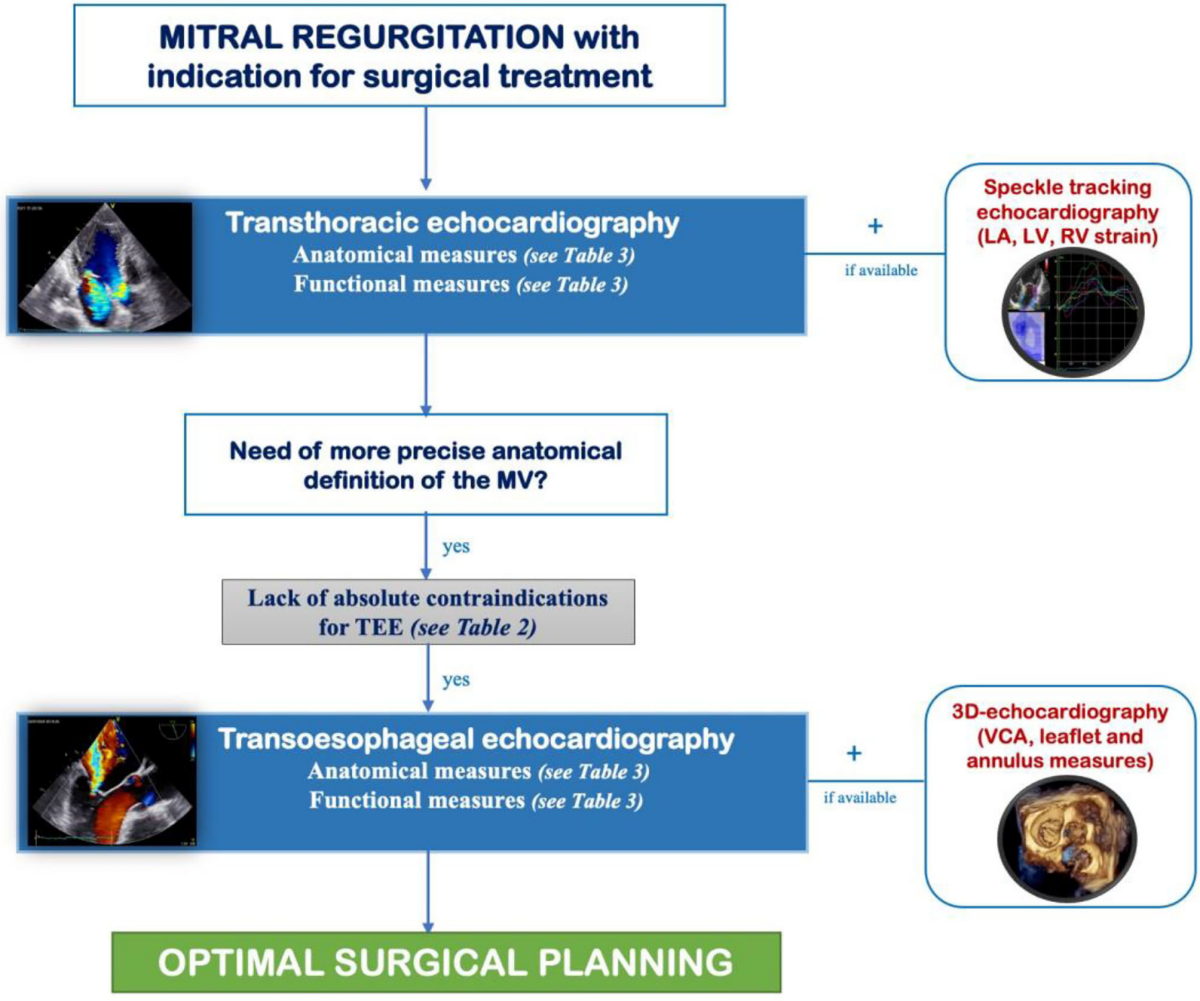
Algorithm to follow for preoperative evaluation of mitral regurgitation in order to promote the collaboration between cardiologist and cardiac surgeon and optimize surgical planning. LA, left atrial; LV, left ventricular; MV, mitral valve; TEE, transesophageal echocardiography; VCA, vena contracta area.

The collaboration between cardiologist and cardiac surgeon is paramount for mitral regurgitation surgical planning and during intervention. Echocardiography plays a pivotal role in the evaluation of mitral regurgitation, and the use of advanced techniques like transesophageal, speckle tracking and 3D echocardiography allows a better definition of anatomical and functional measures in order to optimize surgical planning. This review offers an algorithm to follow (Figure 4), a graphical guide for measurements and a checklist to fulfill to help clinicians in performing echocardiography for preoperative assessment of patients undergoing mitral valve surgery.

## Author Contributions

MP, GM, AS, AD, NG, GB, and AP performed the data search and drafted the manuscript. MC, FD'A, LC, ML, MM, CS, MF, GP, SV, and SM critically revised the draft. All Authors contributed to the conception of this work and approved the final version of the manuscript.

## Conflict of Interest

The authors declare that the research was conducted in the absence of any commercial or financial relationships that could be construed as a potential conflict of interest.

## Supplementary Material

Supplementary Figure 1 Transthoracic and transoesophageal echocardiographic mitral valve anatomic parameters required for the surgical planning of mitral regurgitation. EDD, end-diastolic diameters; IVS, interventricular septum distance; LVOT; left ventricular outflow tract; MV, mitral valve.

Supplementary Figure 2 Example of Vena Contracta Area (VCA) estimation from multi-planar reconstruction of MV in a 3D color Doppler dataset. VCA is obtained by first aligning the blu plane with the vena contracta and then aligning the red and green planes with the jet direction. VCA area is then manually traced in the short axis view (blu plane).
