# Supplementary figures and images for: Two and Three-Dimensional Echocardiography in Primary Mitral Regurgitation: Practical Hints to Optimize the Surgical Planning
Source: Front Cardiovasc Med. 2021 Jul 8;8:706165. doi: 10.3389/fcvm.2021.706165 (PMC8295501; doi:10.3389/fcvm.2021.706165)

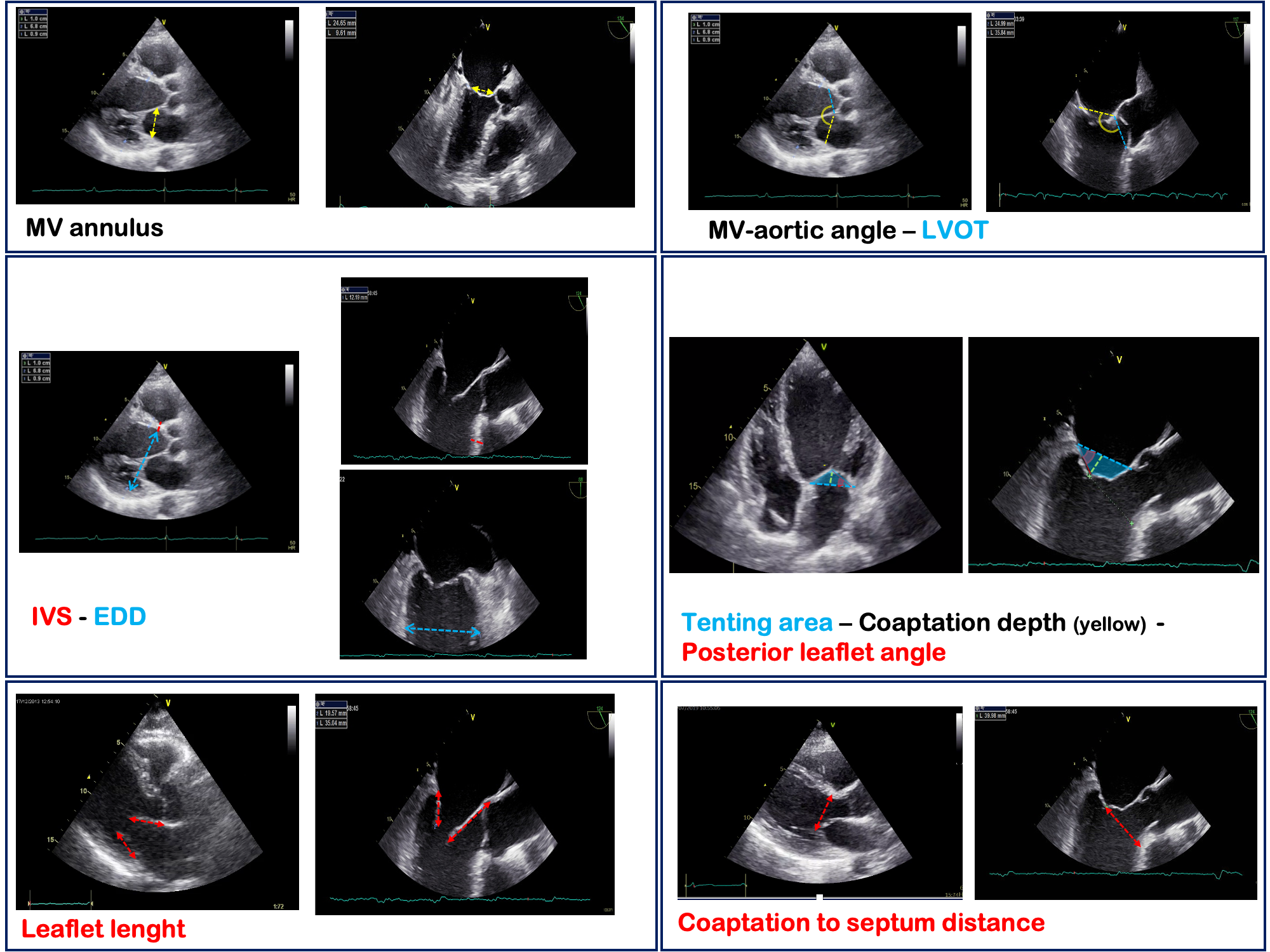

Supplement: Supplementary Figure 1 — Transthoracic and transoesophageal echocardiographic mitral valve anatomic parameters required for the surgical planning of mitral regurgitation. EDD, end-diastolic diameters; IVS, interventricular septum distance; LVOT; left ventricular outflow tract; MV, mitral valve. [file Image_1.TIFF]

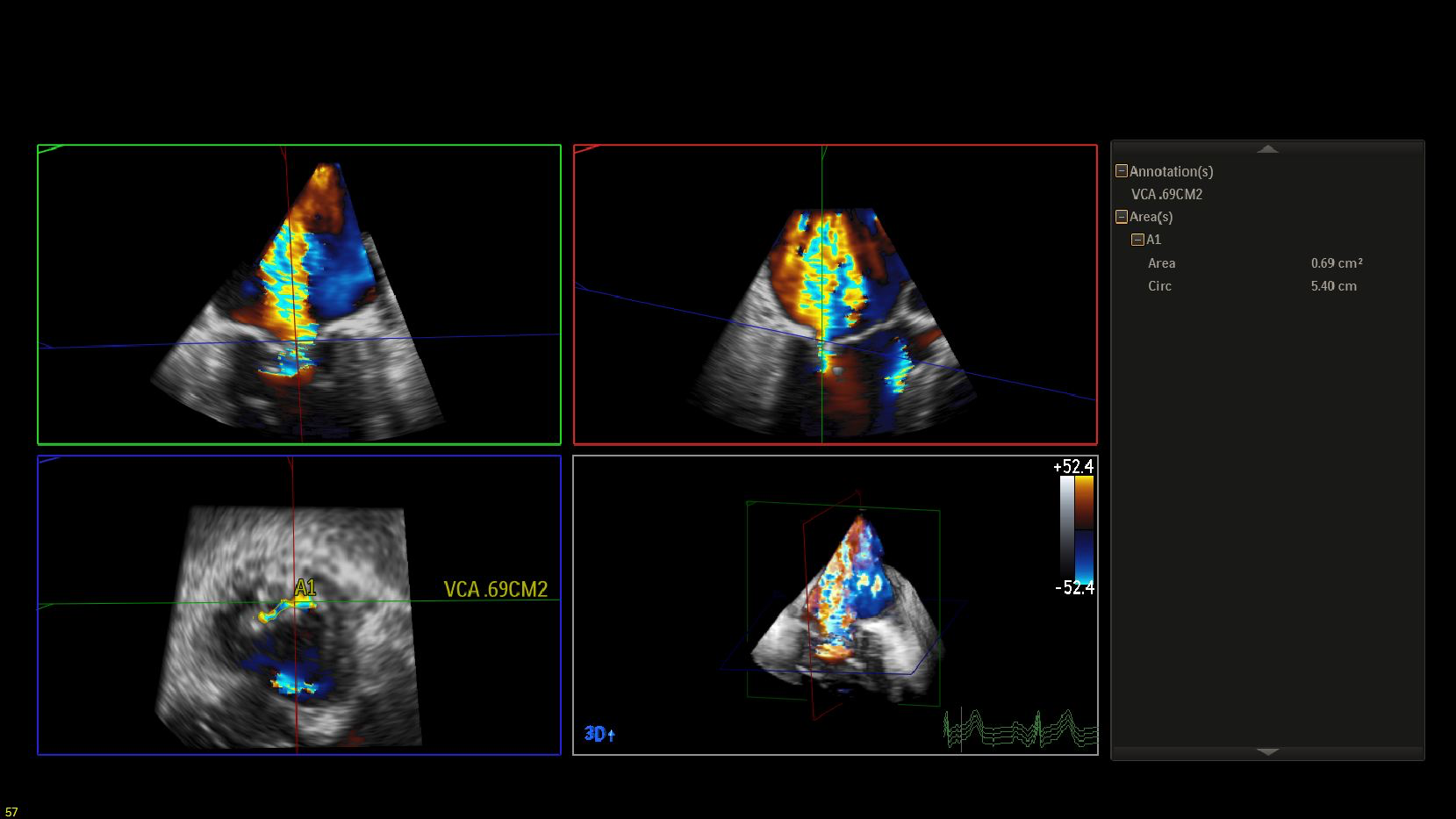

Supplement: Supplementary Figure 2 — Example of Vena Contracta Area (VCA) estimation from multi-planar reconstruction of MV in a 3D color Doppler dataset. VCA is obtained by first aligning the blu plane with the vena contracta and then aligning the red and green planes with the jet direction. VCA area is then manually traced in the short axis view (blu plane). [file Image_2.TIFF]
